# High-throughput screening of cellulase F mutants from multiplexed plasmid sets using an automated plate assay on a functional proteomic robotic workcell

**DOI:** 10.1186/1477-5956-4-10

**Published:** 2006-05-02

**Authors:** Stephen R Hughes, Steven B Riedmuller, Jeffrey A Mertens, Xin-Liang Li, Kenneth M Bischoff, Nasib Qureshi, Michael A Cotta, Philip J Farrelly

**Affiliations:** 1 United States Department of Agriculture (USDA), Agricultural Research Service (ARS), National Center for Agricultural Utilization Research (NCAUR), Bioproducts and Biocatalysis (BBC) Research Unit, 1815 North University Street, Peoria, IL 61604, USA; 2 USDA, ARS, NCAUR, Fermentation Biotechnology (FBT) Research Unit, 1815 North University Street, Peoria, IL 61604, USA; 3 Hudson Control Group, Inc., 10 Stern Avenue, Springfield, NJ 07081, USA

## Abstract

**Background:**

The field of plasmid-based functional proteomics requires the rapid assay of proteins expressed from plasmid libraries. Automation is essential since large sets of mutant open reading frames are being cloned for evaluation. To date no integrated automated platform is available to carry out the entire process including production of plasmid libraries, expression of cloned genes, and functional testing of expressed proteins.

**Results:**

We used a functional proteomic assay in a multiplexed setting on an integrated plasmid-based robotic workcell for high-throughput screening of mutants of cellulase F, an endoglucanase from the anaerobic fungus *Orpinomyces *PC-2. This allowed us to identify plasmids containing optimized clones expressing mutants with improved activity at lower pH. A plasmid library of mutagenized clones of the *celF *gene with targeted variations in the last four codons was constructed by site-directed PCR mutagenesis and transformed into *Escherichia coli*. A robotic picker integrated into the workcell was used to inoculate medium in a 96-well deep well plate, combining the transformants into a multiplexed set in each well, and the plate was incubated on the workcell. Plasmids were prepared from the multiplexed culture on the liquid handler component of the workcell and used for *in vitro *transcription/translation. The multiplexed expressed recombinant proteins were screened for improved activity and stability in an azo-carboxymethylcellulose plate assay. The multiplexed wells containing mutants with improved activity were identified and linked back to the corresponding multiplexed cultures stored in glycerol. Spread plates were prepared from the glycerol stocks and the workcell was used to pick single colonies from the spread plates, prepare plasmid, produce recombinant protein, and assay for activity. The screening assay and subsequent deconvolution of the multiplexed wells resulted in identification of improved CelF mutants and corresponding optimized clones in expression-ready plasmids.

**Conclusion:**

The multiplex method using an integrated automated platform for high-throughput screening in a functional proteomic assay allows rapid identification of plasmids containing optimized clones ready for use in subsequent applications including transformations to produce improved strains or cell lines.

## Background

The field of plasmid-based functional proteomics utilizes full-length cDNA plasmid libraries as a source of clones for evaluation of protein function by *in vitro *transcription/translation or *in vivo *expression methods. Since the libraries are composed of several thousand gene variants, automation of the screening process is essential. To date, a number of automated systems have been developed to assist in screening [1-11], but no single integrated platform exists to carry out the entire process from production of plasmid libraries to functional testing of expressed proteins for large sets of clones. The cell-free system employed by Betton [3] to test the expression level of the protein by *in vitro *transcription/translation from PCR product prior to cloning the gene results in a protein optimized for expression. However, the optimized PCR product identified by this system remains to be isolated and purified manually, then cloned by conventional non-automated methods to evaluate expression of soluble, folded protein *in vivo*. The automated protein preparation platform described by Vinarov and Markley [11] is useful for large-scale production of labeled protein for NMR structural analysis from plasmids obtained via small-scale screening of selected target ORFs for expression levels and solubility. However, the advantage of an automated plasmid-based platform is the ability to obtain a large number of expression-ready plasmid sets that contain optimized clones for automated transformation to produce improved strains or cell lines. Expression of appropriately folded, soluble protein *in vitro *or *in vivo *provides the means to screen for an optimized clone already placed in a plasmid and capable of yielding a functional protein.

We are currently developing an integrated plasmid-based functional proteomic workcell to automate all required tasks: producing cDNA libraries, picking colonies, isolating plasmid DNA, transforming yeast and bacteria, expressing proteins, and performing functional assays. The development of the liquid handler component for automated 96-well plate plasmid preparation on the workcell has been described previously [12]. The integrated automated large-scale liquid handler adaptation described in this paper produces plasmids of sufficient quantity and quality for use in mutagenesis strategies for open reading frame optimization and in generating unique expression-ready cDNA libraries to develop improved strains and cell lines.

The cost of using automated molecular biology techniques in high-throughput recombinant protein screens can be prohibitively expensive. A multiplexed format reduces the time and cost of producing recombinant proteins from large-scale plasmid preparations by decreasing the number of wells to be screened and the amount of reagents needed. Here we present an automated multiplex method made possible by integration of a robotic colony picking component onto an automated workcell platform capable of high-throughput screening of a plasmid library expressing mutants of the endoglucanase CelF to obtain clones giving rise to proteins with improved activity at lower pH and stability at higher temperature. Enzymes such as CelF are capable of digesting plant cell wall polysaccharides and therefore have potential industrial application in breaking down plant biomass for use in fuel ethanol production [13,14]. The multiplex method involves an initial screening of a multiplexed culture that contains a mixture of clones in order to identify those giving rise to *in vitro *expressed improved mutant proteins. Individual clones corresponding to these improved mutants are then isolated by picking single colonies from the multiplexed cultures, producing plasmid DNA from the picked colonies and expressing protein in automated *in vitro *transcription/translation reactions to identify the mutant with improved activity on a plate-based functional assay.

## Results

### Mutagenesis

Targeted mutagenesis of the last four codons of the *celF *gene was initially accomplished by creating a PCR primer set with the reverse primer (anti-sense strand) containing any nucleotide in the first and second positions and a pyrimidine in the last position (NNY). Consequently the sense strand contains the complementary NNR motif where R is a purine. This strategy generates a library of open reading frames that code for 14 of the possible 20 amino acids at each of the last four positions in the CelF mutants, or 14^4 ^= 38,416 possible combinations. Phe, Tyr, His, Asn, Asp, and Cys are not possible since they have only NNY codons. Also, because of the degeneracy of the code, almost every amino acid is represented by several codons (synonyms). In the NNR strategy the amino acids with the most synonyms are leucine and arginine. Although the amino acids with more synonyms have a theoretically greater likelihood of appearing, it is possible that they do not improve the activity of the mutant and may even eliminate activity and thus are not seen in the screening process.

A subsequent large-scale screening run was conducted in which the site-directed PCR mutagenesis strategy was modified to obtain NNY as well as NNR codons in the sense strand. This was accomplished by producing PCR primers with an NNR motif in the anti-sense strand in addition to the primers with the NNY motif. A library of open reading frames is obtained from the NNR primers coding for the six amino acids having only NNY codons and also for the nine other amino acids having both NNY and NNR codons. So the NNY codon strategy gives a total of 15^4 ^= 50,625 possible CelF variants. Together with the 38,416 possible CelF variants from the NNR codon strategy this gives a total of 89,041 theoretically possible CelF variants.

The forward primer contained the CACC sequence in front of the ATG start codon to allow the library of mutagenized clones to be moved into the pENTR D TOPO vector system directionally. The resulting library was transformed into *E. coli *cells, spread onto selective plates and incubated overnight. In the initial NNR screen, approximately 10 to 80 colonies were obtained on each of the 100 plates in this set when each plate was spread with one 25-μL competent cell transformation. In the subsequent large-scale NNR plus NNY screening run, 178 spread plates were prepared from the transformation reactions containing clones with NNY codons and 262 spread plates were prepared from the transformation reactions containing clones with NNR codons. One 25-μL competent cell transformation was used for each spread plate.

### Multiplexed colony picking

**Figure 1:**
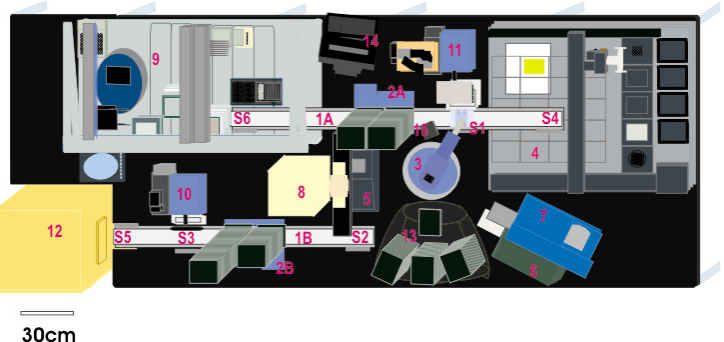
Diagram of the robotic workcell (shown without biocontainment hood). Position #1A: Track 1; #1B: Track 2; #2A: StackLink active stacker (Track 1); #2B: StackLink active stacker (Track 2); #3: 4-axis Hudson Plate Crane EX; #4: BioRad VersArray colony picker/arrayer; #5: PCR thermal cycler with motorized heated lid; #6: UV/VIS plate reader using KC4 software; #7: ABgene 300 plate sealer using foil tape; #8: Brandel RS3000 plate sealer using porous tape; #9: liquid handler with centrifuge; #10: Hudson micro10 filler; #11: Hudson track-based sterile plate aspirator; #12: automated incubator; #13: passive stackers; #14: computer and monitor: #15: barcode reader; #S1-S6: StopLink plate positions.

Colony picking was performed on the automated workcell using the workcell picker (Figure 1 #4) to give multiplexed wells on a 96-well deep well culture plate. The first run entailed picking eight colonies from the spread plates to inoculate each well of an ABgene deep well block containing 1.6 mL of TB KAN 25 medium, giving a total of 768 clones for one plate (8 per well × 96 wells). From the 768 clones screened in this run, five (5) multiplexed cultures with improved activity were identified. The subsequent large-scale run entailed picking 80 colonies to inoculate each well of three 96-well deep well blocks and 4 colonies to inoculate each well of another 96-well block, giving a total of 23,424 clones screened (3 × 96 × 80) plus (96 × 4). Of this total, 9,486 were taken from colonies containing NNY clones and 13,938 were taken from colonies containing NNR clones. The number of clones screened was increased to verify the high-throughput capability of the automated platform.

Integration of the picking operation onto the robotic workcell permits automated multiplexing, which conserves reagents and time. After colony picking was performed, the resulting multiplexed cultures were incubated overnight and processed using the plasmid preparation protocol on the liquid handler of the workcell (Figure 1 #9). Inserts were directionally cloned into pENTR D TOPO using topoisomerase ligation, then into pEXP-1 DEST vector via LR clonase reaction and transformed into TOP 10 *E. coli *cells [12]. The resulting multiplexed plasmid library with mutagenized inserts was used for *in vitro *transcription/translation.

### Screening of multiplexed mutants

**Figure 2:**
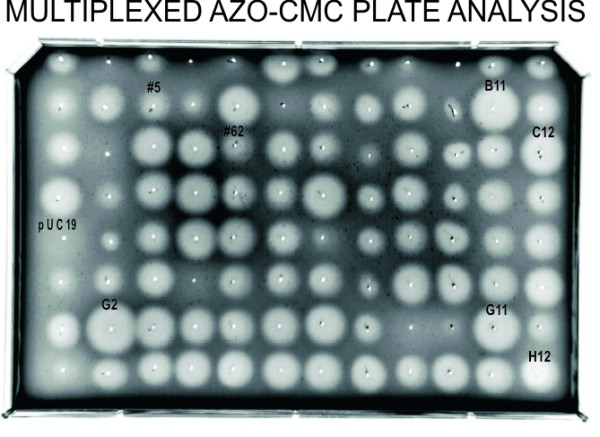
Multiplexed CelF azo-CMC screening plate. *In vitro *expressed protein from multiplexed plasmid preparation was spotted onto agar plate containing azo-CMC (pH 5.8). Wells are identified by the row (letter) and column (number) on the azo-CMC plate. B11, C12, G2, G11 and H12 showed greatest activity and were selected for separation into individual clones (see Figures 6 and 7).

The expressed CelF proteins from the multiplexed transcription/translation reactions were assessed for cellulase activity at several pH levels using an automated azo-CMC plate assay. The results at pH 5.8 showed several cleared zones after incubation for 10 hours at 37°C, indicating cellulase activity (Figure 2). It can be determined from the diameter and intensity of the cleared zones on the plate that protein from wells B11, C12, G2, G11, and H12 had elevated activity at this pH. The negative control placed on the plate (pUC19) in well E1 showed no cellulase activity. Activities of positive controls, wild-type (CelF #5) and an improved mutant (CelF #62) obtained previously [12], are shown in wells B3 and C5, respectively.

**Figure 3:**
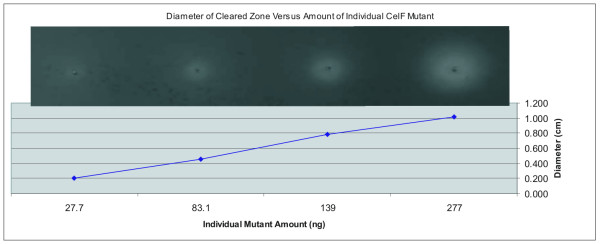
Diameter of cleared zone plotted as a function of increasing concentration of an individual improved CelF mutant. One, 3.0, 5.0 and 10.0 μL of a 27.7 ng/μL solution of F9MP mutant (27.7, 83.1, 139 and 277 ng, respectively) were spotted on an azo-CMC plate at pH 5.8. The resultant cleared zones are pictured in the inset above the graph (incubation at 10 hours, 37°C).

**Figure 4:**
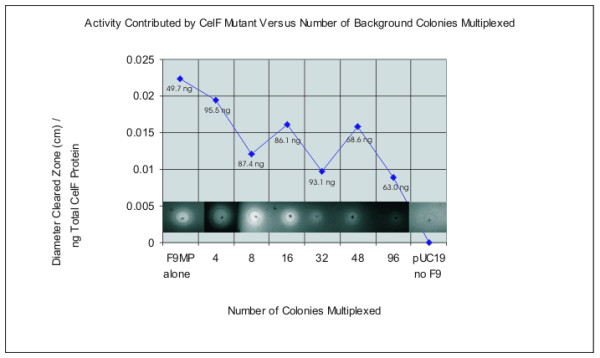
Detection of activity of an individual CelF mutant in a multiplexed mixture as number of colonies multiplexed per well increases. Activity contributed by an individual CelF mutant (F9MP) is plotted as a function of number of background colonies multiplexed as described in Methods. The cleared zones on the azo-CMC plate at pH 5.8 are shown in inset at bottom (10 μL spotted; incubation at 10 hours, 37°C).

Control experiments were performed to determine the effect of increasing amounts of CelF mutants in a multiplexed well or increasing numbers of multiplexed colonies on assay sensitivity. Figure 3 illustrates that as the amount of an individual active CelF mutant spotted on the azo-CMC plate is increased from 27.7 to 277 ng, the activity measured by the diameter of the cleared zone produced on the plate increases in a linear fashion. The cleared zones on the azo-CMC plate at pH 5.8 are shown in the inset at the top of the graph (incubation at 10 hours, 37°C). In addition, if the number of colonies multiplexed in a well is increased relative to an individual CelF mutant, the activity of that mutant is still detectable. Figure 4 depicts the activities obtained when an individual active CelF mutant is added to a mixture containing an increasing number of background colonies multiplexed as described in this paper, from no background colonies (active mutant alone) to 96 background colonies per well. The cleared zones on the azo-CMC plate at pH 5.8 are shown in the inset at the bottom of the graph (incubation at 10 hours, 37°C).

### Identification of individual clones

The multiplexed wells containing mutants with improved activity that were identified on the multiplexed azo-CMC screening plate (Figure 2) were linked back to the corresponding multiplexed cultures stored in glycerol. The identified glycerol stocks were spread on LB AMP 50 plates and single colonies from these plates were picked by the workcell colony picker into a 96-well plate to isolate individual clones from the selected high-activity wells, B11, C12, G2, and G11. The single colonies were picked into TB AMP 50 medium and incubated for 20 hours. In the automated picking process, the colony picker was programmed to select colonies between 0.2 to 10 millimeters in size. This restriction was fixed for a given run but could be reprogrammed if necessary. To determine how this automated picking process compared with manual picking by an individual who has greater leeway in selecting colonies and who may attempt to optimize the variety of colonies being picked, a set of colonies was also picked manually.

**Figure 5:**
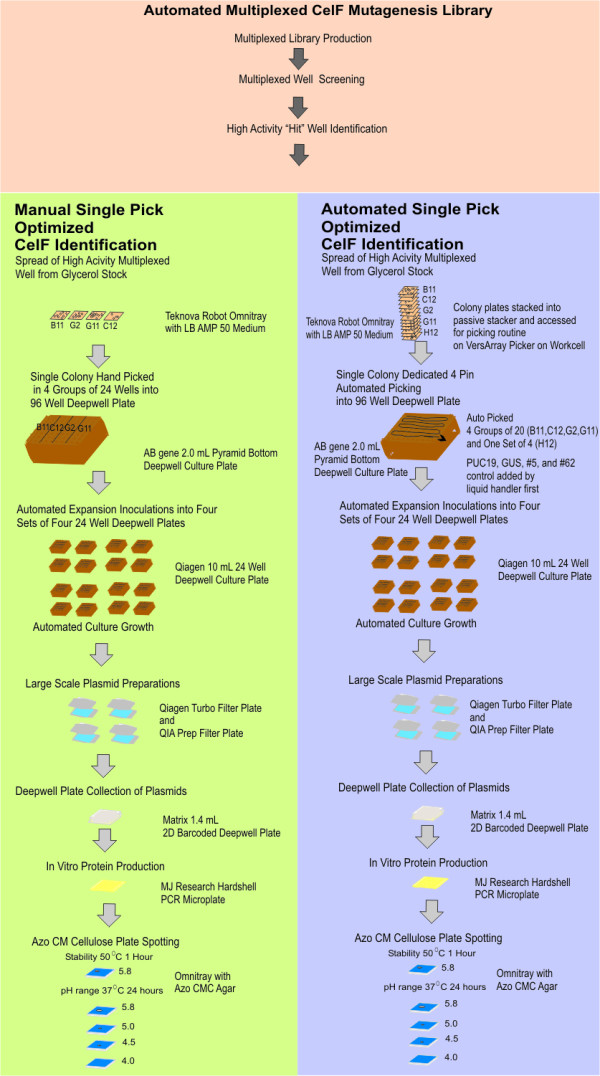
Schematic depicting workcell protocols and plates used in the automated process for separating multiplexed mutagenized clones into individual clones (involving either the manual or the robotic colony picking step).

**Figure 6:**
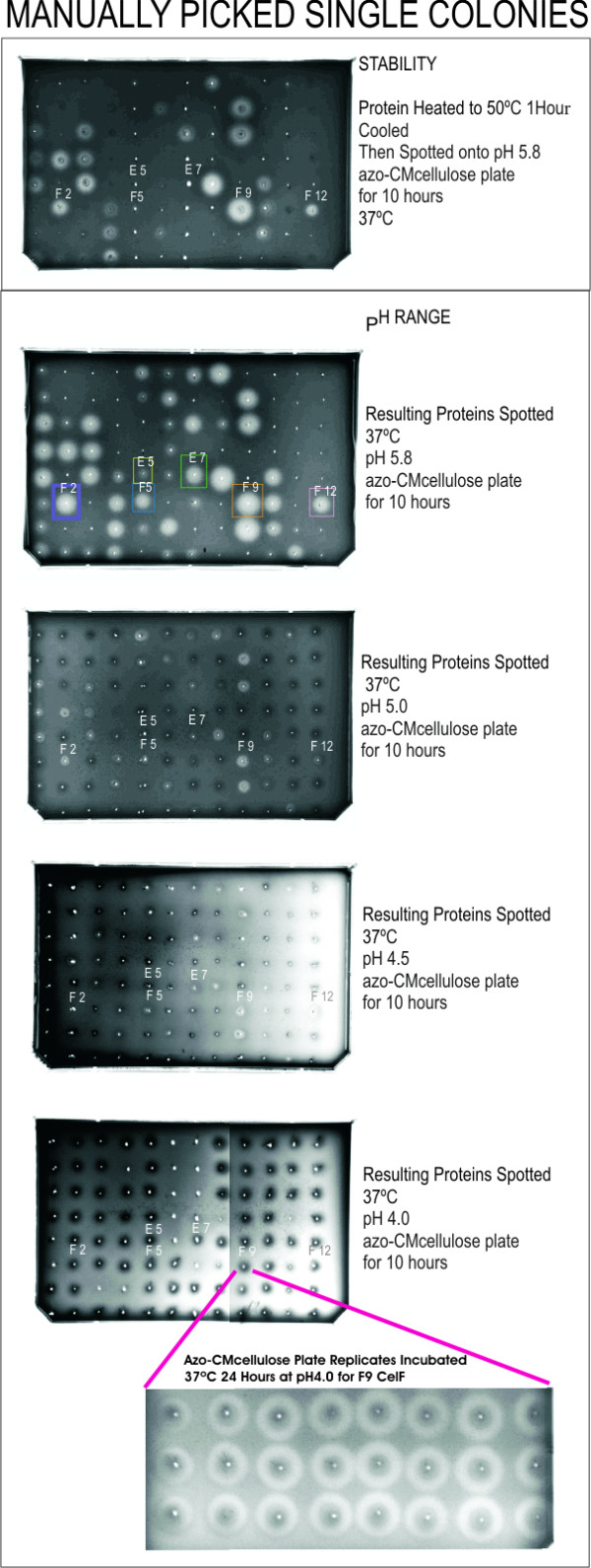
Azo-CMC plate analysis of expressed proteins from manually picked (MP) individual colonies. Individual mutants are identified by row (letter) and column (number) on the plate and by method of picking. Columns 1–3 on each plate were picked from colonies corresponding to multiplexed well B11 (see Figure 2) (high activity variant F2MP is in row F and column 2), columns 4–6 from C12 (high activity variants are E5MP and F5MP), columns 7–9 from G2 (E7MP and F9MP), columns 10–12 from G11 (F12MP). Note that F9MP has the most exceptional activity. Replicates of F9MP activity at pH 4.0 (incubated for 24 hours) are enlarged to better visualize cleared zones. Boxed mutants were selected for Western blot analysis (Figure 8) based on activity and stability

The manual picking was performed using 4 groups of 24 wells on the 96-well plate and taking 24 colonies corresponding to each of the identified wells (process outlined in Figure 5). After expansion of the 96-well plate into four 24-well plates, large-scale plasmid preparation was performed on the liquid handler and the resulting plasmids were eluted into one 96-well Matrix collection plate. The plate was moved to the Bio-Tek UV/VIS microplate reader and the absorbance at 260 nm of each well was adjusted to 1.0 (equivalent to a concentration of 0.05 μg/μL) by diluting with Qiagen EB buffer on the liquid handler deck using the liquid handler pipets and buffer from the cold block on the deck. *In vitro *transcription/translation reactions were performed using the entire plate of plasmids. Protein was spotted on four azo-CMC plates at pH 4.0, 4.5, 5.0, and 5.8. Protein heated to 50°C for 1 hour was spotted on a fifth plate (at pH 5.8). An improved CelF enzyme was isolated from each of the identified multiplexed wells (Figure 6). Individual mutants are identified by row (letter) and column (number) on the plate and by "MP" indicating they were manually picked. Columns 1–3 on each plate were picked from colonies corresponding to multiplexed well B11 (see Figure 2) (high activity variant F2MP boxed in purple is in row F and column 2), columns 4–6 from multiplexed well C12 (high activity variants are E5MP boxed in yellow and F5MP boxed in blue), columns 7–9 from multiplexed well G2 (high activity were E7MP in green and F9MP in orange), columns 10–12 from multiplexed well G11 (F12MP in pink). Clones F2MP, F5MP, F9MP, and F12MP had activity at lower pH than wild-type CelF. Clones F2MP, F9MP, and F12MP were also stable after heating to 50°C for 1 hour. F9MP (boxed), which exhibits the most exceptional activity, was shown to have the same sequence as B9MP, C9MP, and E8MP (all not boxed), which all exhibit activity similar to F9MP. The boxed mutants were selected for Western blot analysis.

**Figure 7:**
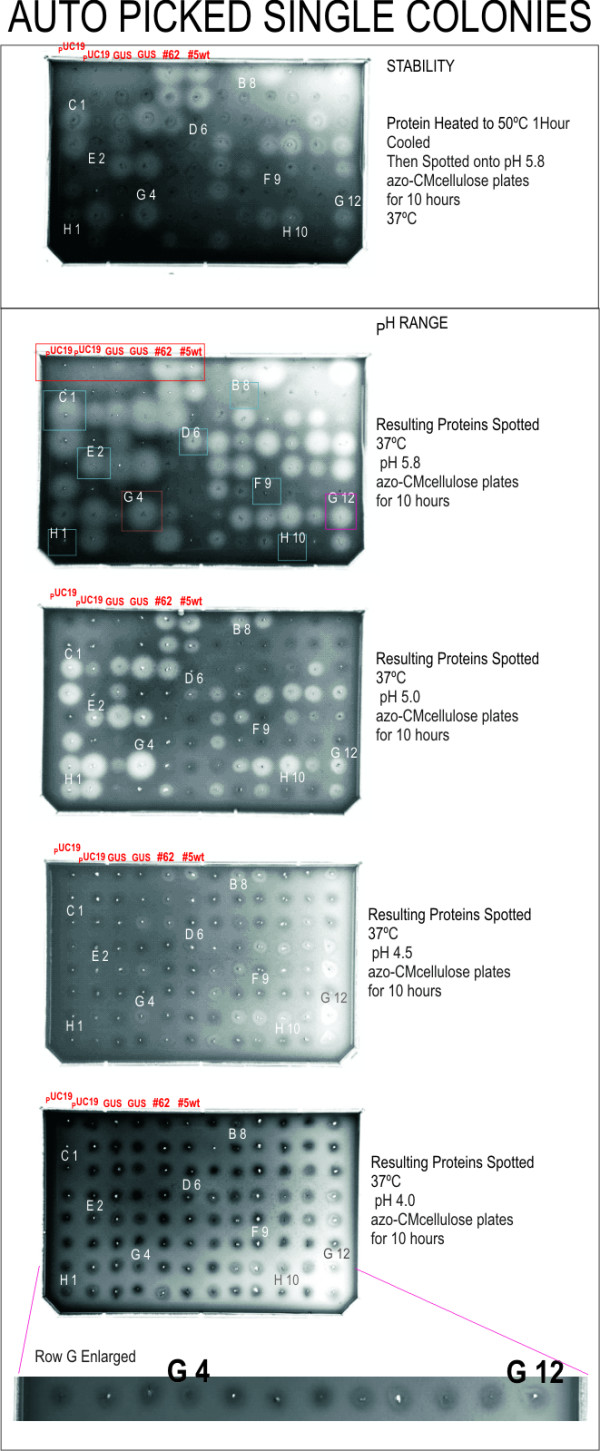
Azo-CMC plate analysis of expressed protein from robotically picked individual colonies. Individual mutants are identified by row (letter) and column (number) on the plate and method of picking (AP – automated picking). Controls are found in the first six locations in the top two rows. Boxed mutants were selected for Western blot analysis (Figure 8) based on activity at low pH and after heating. Colors and letter/number identifiers correspond to those in Figure 8. Row G is enlarged in the inset at bottom to better visualize cleared zones.

The same multiplexed glycerol stock spread plates from which colonies had been picked manually were also used for automated picking into a 96-well plate (see process in Figure 5). The colonies were picked and identified as controls or samples. Expansion of the 96-well plate into the first of the four 24-well plates involved inoculation on the workcell with 84 colonies from five of the identified high-activity multiplexed stocks (B11, C12, G2, G11, H12). Control cultures were placed in 12 of the 24 wells (boxed at top left of Figure 7), including pUC19 (negative control for expression), GUS gene (positive for expression; negative for activity), wild-type CelF #5 and improved mutant CelF #62 [12] (positive controls for activity). The 24-well process was repeated for three more plates. After expansion of the 96-well plate into the four 24-well plates, large-scale plasmid preparation was performed on the liquid handler and the resulting plasmids were combined into a 96-well Matrix collection plate. Plasmid DNA concentrations were adjusted to 0.05 μg/μL using the A_260 _reading from the UV/VIS microplate reader so all clones were present in sufficient quantity for consistent expression, and then *in vitro *transcription/translation reactions were performed for the entire plate of plasmids. The resulting set of recombinant proteins was spotted on azo-CMC plates at pH 4.0, 4.5, 5.0, and 5.8. A fifth plate (at pH 5.8) was spotted with protein heated at 50°C for 1 hour. Results were similar to those for the manual picking procedure and indicated that an improved CelF protein was present in each of the identified multiplexed wells. Two mutants having high activity at lower pH and showing stability at elevated temperature (G4AP boxed in brown, G12AP boxed in red) were identified (Figure 7) in the automated picking process ("AP" indicates clones from this process). Several other CelF variants were seen that had high activity at pH 5.8 and 5.0 but not at pH 4.5 or 4.0 (for example, C1AP and E2AP boxed in aqua). One mutant (H1AP in aqua) had activity only at pH 5.0 and two variants were not stable after heating to 50°C (E2AP and D6AP in aqua). Several clones were aberrant because no insert was detected (B8AP), no plasmid DNA was recovered (H10AP) or an unsuccessful recombinational event occurred (F9AP) as seen when the plasmids were subjected to *Bsr*GI restriction analysis (data not shown).

### Sequences of mutants

Sequences for the mutagenized clones that gave CelF variants with improved activity at lower pH are listed in Table 1. Sequencing results for the manually picked clones identified five unique sequences from the 768 clones originally picked into the multiplexed wells. Clones G12AP and G4AP selected in automated picking had the same sequence as F2MP and F9MP, respectively, from manual picking. The CelF variant (F9MP/G4AP) that had the greatest activity at lower pH and was stable at elevated temperature also contained a point mutation at amino acid 213 in addition to the four amino acid changes at the C-terminus generated by mutagenizing the last four codons of the *celF *gene. A CelF mutant, C7HTS ("HTS" denotes high-throughput screen), that was active at restrictive pH 4.0 and very stable to heating at elevated temperature, with a sequence differing in the last four amino acids from those in the mutants obtained previously, was identified in the large-scale run from the NNY plus NNR mutagenesis strategy. C7HTS also had a premature stop codon, shortening the sequence by one amino acid.

**Table 1:** 

**Clone**		**Plate**	**Sequence of CelF Terminal Codons**							**pH Range**	**50°C, 1 Hour Stability**
Mutagenesis					NNR	NNR	NNR	NNR	*		

WT #5 CONTROL		From single 96-clone screen	**AAT**	**GCT**	**AGA**	**CCA**	**GGA**	**TTC**	**TAA**	5.0–5.8	Moderate
			Asn	Ala	Arg	Pro	Gly	Phe	*		

#62 CONTROL		From single 96-clone screen	**AAT**	**GCT**	**CCA**	**GTA**	**CGG**	**GAA**	**TAA**	5.0–5.8	Moderate
			Asn	Ala	Pro	Val	Arg	Glu	*		

COLONY PICKING			**Variants with High Activity at Low pH**								
										
MAN	AUTO										

**E7**		From G2 well of 768-clone multiplex screen	**AAT**	**GCT**	**TTA**	**TAA**				4.0–5.8	Not Stable
			Asn	Ala	Leu	*					

**F2**	**G12**	From B11 well of 768-clone multiplex screen	**AAT**	**GCT**	**CAA**	**TGG**	**ACA**	**CCA**	**TAA**	4.0–5.8	Moderate
			Asn	Ala	Gln	Trp	Thr	Pro	*		

**F9 **^φ^	**G4 **^φ^	From G2 well of 768-clone multiplex screen	**AAT**	**GCT**	**ATG**	**CCA**	**ACG**	**TTA**	**TAA**	4.0–5.8	Very Stable
			Asn	Ala	Met	Pro	Thr	Leu	*		

**F12**		From G11 well of 768-clone multiplex screen	**AAT**	**GCT**	**CCA**	**TAA**				4.0–5.8	Moderate
			Asn	Ala	Pro	*					

HTS Mutagenesis					NNR/Y	NNR/Y	NNR/Y	NNR/Y	*		

	**C7**	From Plate 2 well of 23,424- clone multiplex screen	**AAT**	**GCT**	**GGG**	**TTA**	**GAA**	**TAG**	**TAA**	4.0–5.8	Very Stable
			Asn	Ala	Gly	Leu	Glu	*	*		

φ An additional Thr to Ser mutation at amino acid position 213 in F9 and G4 clones

**Figure 8:**
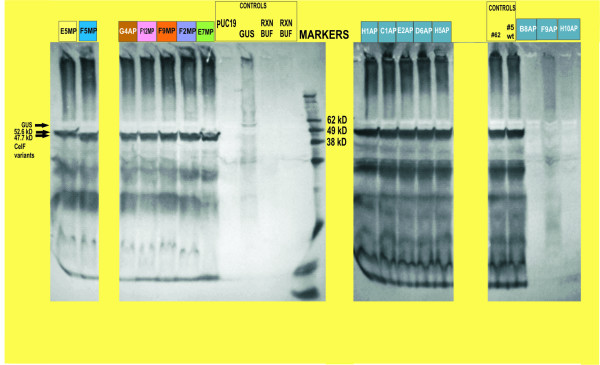
Western blot of *in vitro *expressed proteins generated on the workcell. Variants are identified by row (letter) and column (number) on the azo-CMC plate (Figures 6 and 7) and by method used to pick colonies, either manual (MP) or automated (AP). Colors of boxes above lanes correspond to colors of boxes around cleared zones for those variants on plates in Figures 6 and 7. Wild-type CelF and CelF variants of similar length are at 47.7 kD. E5MP at 52.6 kD had a mutated stop codon resulting in a variant with 54 more amino acids than wild-type. Band for GUS is seen at 60 kD.

Western blot analysis of the expressed proteins is provided in Figure 8. Wild-type CelF (control #5) and variants of the same length as wild-type give a band at 47.7 kD. E5MP in the first lane at 52.6 kD had a mutated stop codon resulting in a CelF variant with 54 more amino acids than wild-type (486 versus 432) (sequence not shown). It had decreased stability after heating and some cellulase activity down to pH 4.5. The G4AP CelF variant, which was shown to have the same sequence as F9MP, demonstrated activity similar to F9MP in that it had greater stability after heating than wild-type CelF and retained cellulase activity down to pH 4.0. Variants F5MP, F12MP, F2MP and E7MP all had activity at lower pH (4.0 or 4.5) but none was as stable after heating as G4AP/F9MP. Variants H1AP, C1AP, E2AP and D6AP demonstrated less cellulase activity than wild-type CelF. Control #62 is an improved CelF variant obtained previously [12]. No cellulase activity and, in fact, no CelF variants were obtained from locations B8AP, F9AP and H10AP. Restriction digest analysis (data not shown) showed primer-dimer insertion, a recombinational event or no plasmid was present in those cases, respectively.

**Figure 9:**
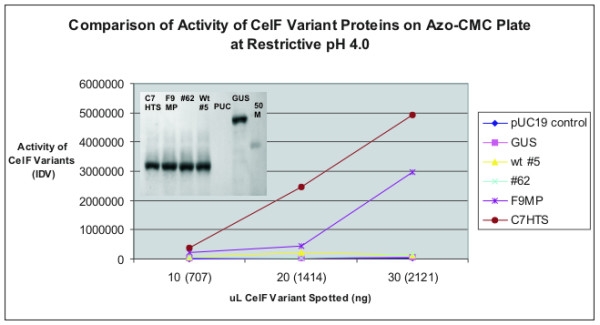
Comparison of activities of wild-type CelF and CelF mutant proteins in integrated density values (IDV) as a function of concentration on azo-CMC plate at pH 4.0 incubated 10 hours at 37°C. Inset is Western blot analysis (with 50 kD marker) showing all proteins are present at the same concentration.

Figure 9 provides a comparison of the activity of wild-type CelF and CelF mutants, #62, F9MP, and C7HTS, at pH 4.0. C7HTS from the large-scale screen demonstrates the greatest activity compared with all other clones obtained to date. The Western blot analysis in the inset at the top shows that the improved CelF variants C7HTS, F9MP and #62 were present at the same concentration for all comparisons.

## Discussion

The multiplex method described here using an automated plasmid-based workcell allows large-scale expression and rapid functional analysis of proteins using an azo-CMC plate assay to evaluate enzyme activity. Integration of the robotic colony picker onto the automated workcell platform permits the complete process from colony production to protein analysis to be performed on a single robotic platform. Our first application of this integrated workcell was the screening of a library of mutagenized *celF *clones. The *celF *cDNA [15,16] used for the mutagenesis strategy was initially provided in the pBlueScript^® ^vector with a polyhistidine (6 × His) tag at the amino terminus for purification by binding to Ni beads, and containing a regular T7 promoter for *in vivo *expression in bacteria. Cellulase activity was evaluated using a liquid-based p-nitrophenol assay. Our intention was to use the Invitrogen Gateway^® ^recombinational cloning system to completely automate plasmid production and process a larger number of clones. A modified T7 promoter was employed for optimum *in vitro *transcription/translation and, in addition, the 6 × His tag was placed at the carboxy terminus to purify primarily full-length expressed proteins. As an accommodation for the automated platform, a plate-based azo-CMC assay was developed instead of the liquid-based assay. However, addition of the 6 × His tag to the carboxy terminus eliminated cellulase activity in the liquid-based p-nitrophenol assay. Moving the tag back to the amino terminus gave activity on the azo-CMC plate assay, suggesting that the carboxy terminus of CelF may play an important functional role. Efforts were therefore focused on modifying the last four amino acids at this end of the enzyme to maximize the effect on its activity.

We reasoned that mutation of four codons should provide a large enough number of variants to obtain some with improved activity but still give a manageable number of clones that could be handled in a realistic time on the automated platform. The NNR mutagenesis strategy targeted to the last four amino acids at the carboxy terminus of CelF and providing codons for 14 amino acids yielded sufficient viable variants with improved activity so that the initial run in which eight colonies were multiplexed per well could identify five unique variants with improved activity. If no optimized variant had been obtained, the number of colonies multiplexed per well could have been increased and/or the automated picking process could have been repeated for rapid screening on the automated platform. Although only 768 of the 38,416 theoretically possible NNR mutagenized *celF *clones picked in the initial run were screened (2%), several improved clones were obtained. In the subsequent NNR plus NNY mutagenesis run, 23,424 of 89,041 or 26% of the theoretically possible clones, 19% of the NNY clones and 36% of the NNR clones, were screened. This strategy yielded at least one additional CelF variant with exceptional activity. The number of viable variants obtained and of colonies that must be multiplexed will depend on a variety of factors, including the gene being mutagenized and the mutagenesis strategy.

Sufficient plasmid can be produced by the workcell to conduct repetitive protein expression reactions and functional assays to rapidly identify multiplexed wells containing CelF mutants with improved characteristics over wild-type CelF. These multiplexed wells are then deconvoluted to identify individual clones. The assay for analysis of the expressed proteins was developed keeping in mind that a rapid qualitative measurement of the activity of the recombinant CelF mutants is required. We chose an agar-based gel-diffusion assay containing azo-CMC that was readily adapted for high-throughput screening on a robotic platform.

The need for sufficient protein for the functional proteomic assays made it necessary to configure the workcell so it produced highly concentrated and abundant plasmid DNA. This was achieved by making quadruplicate preparations in 24-well plates with 5 mL of medium in each well followed by elution and collection into one 96-well plate to give a final volume of 20 mL. The four plates that were eluted into one 2D Matrix collection plate gave more than four times the amount of plasmid previously observed for one plate when using 1.6 mL of cultures [12]. As reported previously, one 96-well Matrix plate gave 5.35 μg per well [12], so for four plates the expected yield would be over 21.4 μg. In the process described in this paper, an average of 31.1 μg per well was obtained (data not shown). However, the amount of starting culture taken here was 20 mL, while in the earlier experiment the amount of starting culture was 1.6 mL per plate (or 6.4 mL if four plates were used). Quantities greater than the amount used here exceed the capacity of the filter plates causing the wells to become blocked so the wash solution cannot be properly pulled through and the plasmid quality deteriorates.

Linking the multiplexed wells with improved CelF activity back to the multiplexed glycerol stocks and isolating the individual clones from the glycerol stock spread plates are carried out in a completely automated fashion on the workcell. For purposes of comparison, the initial colony-picking operation was performed both manually and on the workcell. Two improved mutants, F2MP and F9MP, isolated by the manual picking process had the same sequence as G12AP and G4AP, respectively, isolated by the automated picking process. All the high-activity wells that were separated out at the colony level gave rise to at least one mutant with increased activity (F2MP/G12AP, F5MP, F9MP/G4AP, F12MP). We initially selected eight picks for each multiplexed well based on the expectation that a large number of different mutants would be obtained. If too many picks were combined, identification of the individual mutants with increased activity might be difficult. With eight picks no multiplexed high-activity wells were seen in which more than one variant contributed to the increased activity, indicating eight picks per well was enough to give optimized mutants of CelF while being a manageable number for testing the protocols on the automated platform. For other genes, no optimized mutants might be found with eight picks per well. In such cases the number of picks can be increased as needed on the automated platform.

The control experiment, in which the amount of a known variant (F9MP) with improved activity was increased and the activity determined, demonstrates that in the range of concentrations encountered, the activity on the azo-CMC plate increases in a linear fashion as the amount of CelF variant increases. In addition, if the number of colonies multiplexed is increased relative to the amount of an individual CelF variant in a multiplexed well, the activity of a single variant can still be detected even if as many as 96 colonies are multiplexed in one well.

*In vitro *transcription/translation is expected to express close to the theoretical maximum number of clones from the mutagenesis strategy when the automated process is run repetitively since it avoids the issues of folding, inclusion body formation, or cell poisoning often found with *in vivo *expression. When all plasmids in one group of 24 from the manual picking process were sequenced (from multiplexed well C12), five unique sequences were found among the 24 plasmids (three of the eight picked clones were duplicates). Duplication is quite possible since several clones were picked from the same transformation plate. It is possible that some of the eight inserts picked at the pENTR D TOPO stage were not cloned recombinationally into the pEXP-1 DEST expression plasmid. Also some of the pEXP-1 DEST clones might not have transformed into the TOP 10 propagation cells. Transformation into TOP 10 chemically competent *E. coli *cells without f' pilus avoids phage contamination and propagates plasmids without expression from the T7 promoter so expression of the clones does not affect survival of *E. coli *cells. Competition might also cause loss of some inserts. *Bs*rGI restriction analysis was performed on the sets of plasmid to determine the number of full-length inserts present. It was found that from the 180 clones picked from the spread plates (manual and automated picking combined), 143 or 79% had a full-length or nearly full-length insert (data not shown). Although there is some insert loss, the automated workcell has the ability to screen large numbers of mutagenized clones.

The quantity of protein produced in the *in vitro *transcription/translation reaction appears to be less dependent on the amount of plasmid than on the amount of reagent mix used, as seen in the control well for the automated run. A statistical evaluation (using manually picked clone F9MP in all 96 wells) showed less variation in the protein amount (COV = 13%; n = 24; avg = 1358 ng/25 μL reaction) than the variation in the amount of plasmid (COV = 22%; n = 96; avg = 31.1 μg/well) from which it was expressed (see inset at bottom of Figure 6). Protein assessment showed the protein produced from a given well was fairly consistent with the other 23 control wells examined (COV for activity was 12%). It is possible that a maximum amount of RNA message is produced from a threshold level of plasmid. This suggests it is important to keep the amount of reagent constant at a level sufficient for consistent expression in the *in vitro *expression reactions, while keeping the plasmid at the threshold level.

Randomizing the sequence at the end of the insert coding for the last four amino acid positions at the carboxy terminus of CelF provided mutants with a range of activities. F2MP/G12AP was an interesting variant of CelF with activity down to pH 4.0 and also higher activity at pH 5.8 than that of wild-type CelF. It had the same moderate stability as wild type after being heated at 50°C for 1 hour. The sequence for this variant reveals the last four amino acids are Gln Trp Thr Pro. F9MP/G4AP was the most improved clone selected in the initial screen. It demonstrated high activity down to pH 4.0 with much higher stability than wild-type CelF after heating to 50°C for 1 hour. Further biochemical and crystallographic investigation of this clone is underway. The clone also had a conservative point mutation at amino acid 213, specifically a Thr to Ser change, in addition to the change in the last four amino acids to Met Pro Thr Leu resulting from the NNR mutagenesis strategy. This might help to account for its activity at lower pH and its stability after heating. These two mutant CelF enzymes of interest (F2MP/G12AP and F9MP/G4AP) maintained the same total number of amino acids (412) and both contained a Pro residue in the last four amino acids similar to the wild-type CelF sequence. The point mutation probably resulted from lack of fidelity of the Taq DNA polymerase in the PCR reaction.

Two clones that ended in a truncated sequence, E7MP and F12MP, had activity similar to wild type at the pH levels tested, but had decreased thermal stability. The carboxy terminus amino acid sequence for E7MP was truncated at Leu by insertion of a stop codon, causing the last three amino acids to be missing. The other truncated mutant F12MP was moderately stable and ended in Pro. This CelF mutant, containing only one amino acid of the last four, demonstrated activity down to pH 4.0. Most of the mutants with improved thermal stability and pH range contained a proline among the last four amino acid positions. In the cellulase tunnel in the CelF structure that has been described [17,18] the presence of proline among the last four amino acids in the enzyme may facilitate substrate access. Our results suggest that the C-terminus is important to both thermal stability and activity at lower pH.

Three clones (E5MP, E7MP, F12MP) were found whose sequence contained a mutated TAA stop codon or in which additions of nucleotides to the ORF led to longer proteins, as seen in the Western blot (Figure 8; E5MP). This may have been the result of errors in the oligomers. None of these mutants had better thermal stability or higher activity at any pH than wild-type CelF. This was in accordance with the observation that adding a tag to the C-terminus of CelF caused loss of cellulase activity.

The sequence of the CelF mutant with most improved activity obtained to date, C7HTS, isolated from the NNY plus NNR strategy in the 23,424-clone run also contained amino acid substitutions in the last four positions that resulted from NNR codons. No high-activity mutants identified to date in this screen had amino acids in the last four positions resulting from NNY codons. Although truncated, the C7HTS mutant had the highest activity on the azo-CMC assay. We are continuing to investigate amino acid substitutions resulting from the NNR plus NNY strategy.

## Conclusion

It was possible to identify and isolate individual improved *celF *open reading frames from colonies picked from multiplexed sets of mutagenized clones of the *celF *gene. For the *celF *gene, multiplexing of eight colonies per well was sufficient to obtain five (5) unique improved CelF mutants. Screening was performed in high-throughput on a robotic workcell using an automated azo-CMC plate assay to analyze the *in vitro *expressed proteins and identify optimized clones from the mutagenized libraries. *In vitro *expression for this gene avoided the issues frequently seen with *in vivo *expression. The combined operations of colony picking from multiplexed plates, large-scale plasmid preparation, *in vitro *transcription/translation, and azo-CMC plate protein assay, followed by isolation and analysis of individual clones, were conducted on an automated platform to screen CelF mutants resulting from a targeted clone mutagenesis strategy. For comparison purposes, both manual and automated colony picking operations were conducted. Both led to identification of the same high-activity thermally stable clones from the multiplexed screening plate of CelF mutants. This demonstrates that standard molecular biological techniques can be applied to high-throughput functional proteomic analysis when placed on a plasmid-based robotic workcell using the automated operations described here for identification and isolation of an improved clone from a multiplexed mixture of clones. The multiplex method using an integrated automated platform for high-throughput screening in a functional proteomic assay allows rapid identification of plasmids containing optimized clones ready for use in subsequent applications including transformations to produce improved strains or cell lines.

## Methods

### Plasmid-based proteomic workcell

The ProLink Express™ workcell (Figure 1) was assembled by Hudson Control Group, Inc., Springfield, NJ. This fully integrated automated plasmid-based functional proteomic workcell is capable of performing the following operations: 1) plate filling, 2) colony picking and arraying in multiplexed wells, 3) large-scale plasmid preparation, 4) protein expression, and 5) functional protein assays. Protocols for using the liquid handler component of the workcell for plasmid preparation and plasmid analysis have been described previously [12]. Plasmids were analyzed on the workcell, by adding samples from the Matrix collection plate to a UV optically clear 96-well VWR microplate, and measuring the absorbance at 230 nm, 260 nm and 280 nm in the Bio-Tek UV/VIS microplate reader (Figure 1 #6). Additional spectrographic analysis of plasmid samples was performed using the NanoDrop ND-1000 fiber optic spectrophotometer (NanoDrop Technologies) to develop a factor to adjust for variances in the height of the readings taken by the UV/VIS microplate reader. Plasmids were digested with *Bsr*GI and the plasmid preparations were run on 2% agarose gels with ethidium bromide (Sigma) added to visualize the DNA. The gel was photographed with an Alpha Imager 3400 using software version IS-3400 V 3.1.2 for Windows 2000/XP. Plasmids were sequenced using a MJ Tetrad PCR machine model PTC 225 with the Big-Dye Terminator V3.1 (Applied Biosystems) according to the manufacturer's instructions and run on an ABI 3730 DNA Analyzer (Applied Biosystems).

### Preparation of cellulase F mutagenesis library

A mutagenized *celF *library was generated as described previously [12] using *celF *cDNA [15,16] (GenBank Accession Number U97154) from the anaerobic fungus *Orpinomyces *PC-2 as template. In addition, NNY libraries were generated in a similar fashion using primers with NNR in the anti-sense strand. Methods for topoisomerase ligation of mutagenized PCR products into pENTR D TOPO, transformation into TOP 10 *E. coli *cells, subsequent plasmid preparation, recombinationally subcloning inserts into pEXP-1 DEST using LR clonase, transformation into *E. coli *cells and plasmid preparation for use in *in vitro *transcription/translation were described previously [12]. Here subcloning into pEXP-1 DEST, transformation into *E. coli *cells and plasmid preparation for *in vitro *transcription/translation were performed in an automated fashion on the workcell.

### *In vitro *expression of cellulase F on multiplexed plasmid plate

A 5-μL aliquot of the pEXP-1 DEST plasmids prepared on the liquid handler was used for *in vitro *transcription/translation reactions with *E. coli *lysate containing T7 polymerase (RTS 100 Kit; Roche Applied Science) to generate recombinant protein. Half reactions at 25 μL were performed instead of the full reaction at 50 μL described in the directions for the kit. Reactions were incubated for 10 hours at 30°C. Nine (9) μL of the samples were run in Western blot analysis (as described previously [12]) and 5 μL of the reactions were spotted (unless otherwise noted in the figure legends) for azo-CMC plate analysis using the assay described previously [12]. Relative activity of the enzyme was scored by measurement of the diameter or integrated density values of the cleared zone.

### Isolation of individual colonies from multiplexed cultures

Those multiplexed wells identified by the screening azo-CMC plate assay as containing mutant(s) with enhanced glucanase activity were linked back to the glycerol stocks containing the pEXP-1 DEST plasmids that gave rise to these wells. Samples of the identified stocks were inoculated onto LB AMP 50 86 × 128 mm spread plates capable of being handled by the robot. The resulting individual colonies, representing all the colonies that were inoculated into each of the multiplexed wells, were either picked manually or automatically for subsequent analysis.

### Manual picking of colonies into 96-well plate

Colonies from spread plates for the identified multiplexed wells were picked manually and used to inoculate TB AMP 50 medium in a 96-well plate. The inoculated plate was manually sealed with gas-permeable tape, taken to the workcell shaker and incubated for 24 hours at 37°C and 150 rpm.

### Automated picking of colonies into 96-well plate

The spread plates containing the colonies from the multiplex cultures were placed onto the workcell in the passive stacker (Figure 1 #13). An ABgene 96-well deep well plate was loaded into the active stacker, a 4-liter carboy containing 2 liters of TB AMP 50 medium was connected to the Hudson micro10 filler (Figure 1 #10), and the automated protocol for picking colonies into the 96-well plate was initiated. The ABgene 96-well deep well plate was sent from the Track 2 active stacker (Figure 1 #2B) to the Hudson micro10 filler (position S3) and each well filled with 1.6 mL of TB AMP 50 medium and the plate moved to the Track 2 Plate Crane StopLink position S2. The plate was moved by the plate crane (Figure 1 #3) from position S2 to S1 and then sent to S6 for inoculation with each of the four control cultures from the cold tube rack on the liquid handler deck (Figure 1 #9) into the first 6 wells in the two upper left hand rows in blocks of two or four using the Hudson liquid handler. After the controls were added, the plate was sent via a Hudson TrackLink (Figure 1 #1A) into the deck space of the picker in StopLink position S4. From this location the gripper tool of the picker (Figure 1 #4) moved the deep well plate with medium into the picker destination location and removed the lid to receive colonies. Picking was performed using a Hudson SoftLinx adapted routine of the BioRad VersArray picker integrated into the workcell. The spread plates for the identified wells were moved from the Hudson passive stacker (Figure 1 #13) by the plate crane (Figure 1 #3) to S1. SoftLinx software from Hudson was used to identify spread plate wells as the plates were moved onto the deck of the picker passing the bar code reader on Track 1 (Figure 1 #1A and #15). The plate was then moved to S4 where the gripper tool on the picker took the colony plate into the lighted area of the picker deck (Figure 1 #4). The CCD camera photographed the spread plate in the lighted area to record a digital image for capture and recognition of stereotactic coordinates for all colonies of the programmed size range. The colonies were picked using four dedicated pins of the 16-pin picking head (Figure 1 #4) and inoculated, four colonies at a time, in an S-shaped pattern into the 96-well plate, randomly taking 20 colonies from each of the first four spread plates and 4 colonies from the last spread plate. The inoculated plate was taken from the destination location to S1, picked up, placed into the Brandel RS3000 porous tape sealer (Figure 1 #8) and sealed with gas-permeable tape. The plate was moved to S2 and loaded into the Liconic incubator (Figure 1 #12) at the modified StopLink S5 and incubated for 30 hours at 37°C and 600 rpm. Glycerol stocks and long-term storage cards were prepared in the work area of the liquid handler (Figure 1 #9) from these cultures after incubation.

### Automated inoculation from 96-well plate into four 24-well plates in quadruplicate (4 × 4 × 24 wells)

The protocol was carried out with a modified SoftLinx integration routine (Figure 1 #14). Qiagen 24-well deep well plates were loaded into the active stacker (Figure 1 #2B), a 4-liter sterile carboy containing 2 liters of TB AMP 50 medium was attached to the filler (Figure 1 #10) with sterile tubing, and the automated protocol for liquid culture inoculation into 24-well plates was initiated. The 24-well deep well plates were sent from the Track 2 active stacker (Figure 1 #2B) to the micro10 filler (position S3) and each well filled with 5 mL of TB AMP 50 medium. The plates were moved from S3 to Track 2 position S2 then to Track 1 position S1 using the Plate Crane EX (Figure 1 #3) and sent via a Hudson TrackLink to position S6 and then into the deck of the liquid handler unit (Figure 1 #9) of the workcell using the gripper arm of the liquid handler. Four 24-well plates at a time were inoculated with 20 μL culture in each well in the image of the 96-well plate four times to give quadruplicate sets of four 24-well plates representing the controls and the 84 colonies picked. The inoculated plates were taken to position S6 then moved to S1 on Track 1, picked up by the plate crane, placed into the porous tape sealer (Brandel RS3000) (Figure 1 #8) on the workcell and each plate sealed with gas-permeable tape. All of the plates were moved to position S2 on Track 2 and then to S6 and into the Liconic incubator for 30 hours at 37°C and 600 rpm. Large-scale plasmid preparation was then carried out on the liquid handler as described previously [12]. These plasmids were eluted on the workcell into one Matrix 2D bar-coded collection plate, samples placed in a UV/VIS microplate (Figure 1 #6), and the concentrations adjusted for consistent expression.

### Automated *in vitro *protein production

MJ plates and UV/VIS optically clear standard microplates were placed into the active stacker (Figure 1 #2A). Protein reagent was filled into Matrix 2D bar-coded tubes and placed into the cold reagent position on the liquid handler (Figure 1 #9) for holding at 2°C. The protocol for protein production adapted from SoftLinx, accessing XAP routines for reaction set up and spotting (Figure 1 #14), was initiated. Protein reactions were prepared from plates of plasmid on the deck of the liquid handler, then a new MJ plate was moved from the active stacker to S6 on the liquid handler deck and transported by the liquid handler gripper to the cold block for reagent additions. The reagents were mixed by aspiration and the plate moved to S6 then to position S1 for transport by the plate crane into the ABgene 300 foil heat sealer (Figure 1 #7) and finally into the PCR thermal cycler (Figure 1 #5). After the plasmids were obtained using the automated plasmid preparation protocol for large-scale plasmid preparation, 5-μL aliquots of the pEXP-1 DEST mutagenized library plasmids were used for *in vitro *transcription/translation reactions with the *E. coli *lysate containing T7 polymerase from an RTS 100 Kit (Roche Applied Science) to generate recombinant protein. Half reactions at 25 μL (5 μL of plasmid from plasmid preparations were used instead of 10 μL) were performed instead of the full 50-μL reaction described in the directions for the kit. This half reaction supplied enough expressed protein for cellulase assays and for Western blot analysis. Reactions were incubated according to directions for 10 hours at 30°C in the PCR thermal cycler after sealing with foil tape. The temperature was then held at 4°C until azo-CMC plate spotting could begin.

### Temperature and pH evaluations of expressed protein using automated azo-CMC plate assay

For pH activity profiles, four azo-CMC plates at varying pH (5.8, 5.0, 4.5, and 4.0) were loaded into the passive stacker (Figure 1 #13), de-lidded by the gripper arm of the plate crane (Figure 1 #3) and moved to S1, then to S6 into the working area of the liquid handler (Figure 1 #9) for the automated azo-CMC plate assay. The plate crane moved the 96-well hardshell plate containing the *in vitro *produced protein from the PCR thermal cycler (Figure 1 #5) to S1. From this position the plate was taken to S6 so that the liquid handler gripper could place it into the cold plate position on the deck, then into the jig position for piercing and finally back to the cold position on the deck. A SoftLinx-initiated XAP protocol was used to spot protein on the azo-CMC plates for the assay using the pipet arm of the liquid handler. Plates were moved to S6 with the gripper, then sent to S1 to be picked up by the plate crane and placed on S2. The plates were moved to S5 for loading into the incubator. For evaluation of temperature stability, the hardshell plate with protein was moved to the PCR thermal cycler (Figure 1 #5), incubated at 50°C for one hour then moved back to the cold position on the liquid handler deck. A new pH 5.8 azo-CMC plate was moved into the working area, spotted with the heated proteins, and moved to the Liconic incubator with the other azo-CMC plates. All the plates were incubated at 37°C for 10 hours. Plates were moved to the out stack on the passive stacker and photographed using the Alpha Innotech 3400 digital imaging station.

## Competing interests

The author(s) declare that they have no competing interests.

## Authors' contributions

SRH designed the workcell, outlined workcell protocols, and planned molecular biology strategy. SBR and PJF designed and assembled the workcell and integrated the workcell protocols. XLL provided *celF *cDNA from *Orpinomyces *PC-2. JM determined plasmid concentrations. Data analyses and technical assistance were provided by SRH, JM, NQ, XLL, KB, and MC.
